# Constructing collective identity through Theravāda Buddhism: religious memory, monastic authority, and sacred infrastructure among the Bulang community in Southwest China

**DOI:** 10.3389/fsoc.2026.1775508

**Published:** 2026-08-20

**Authors:** Wenen Yan, Yaoping Liu

**Affiliations:** Department of Global Buddhism, Institute of Science Innovation and Culture, Rajamangala University of Technology Krungthep, Bangkok, Thailand

**Keywords:** Bulang ethnic community, collective identity, religious infrastructure, sacred biography, Sangha, Southwest China, Theravāda Buddhism

## Abstract

**Introduction:**

Theravāda Buddhism plays a central role in the cultural and religious life of several ethnic communities in Southwest China, yet the ways minority communities mobilize Buddhism to construct collective identity remain insufficiently explored.

**Methods:**

This study used ethnographic fieldwork, participant observation, and semistructured interviews with ten purposively selected participants in Laoman’e village, Xishuangbanna, Yunnan.

**Results:**

The findings show that Buddhist architecture, including temples, stupas, shrines, monasteries, and ritual areas, functions not only as places of worship but also as symbols of Bulang Buddhist legitimacy and cultural continuity. The sacred biography of Phra Somdet Aggamuni, locally also remembered as Meghamuni, operates as a form of soft religious infrastructure through which spiritual stories and moral values are transmitted across generations. The continuity of Bulang Buddhism is sustained through the mutual engagement of monks, elders, lay Buddhists, women, and youth in rituals, donations, storytelling, temple renovation, and ceremonies such as Kaṭhina.

**Discussion:**

The study demonstrates that Theravāda Buddhism in the Bulang community is not merely inherited as tradition; it is transformed, actualized, retold, and performed as a living system of collective identity.

## Introduction

The study of Buddhism in Asia has often been shaped by dominant religious, national, and institutional narratives, while smaller ethnoreligious communities have received comparatively limited scholarly attention. It becomes even more obvious when discussions revolve around Theravāda Buddhism because most of them focus on major Buddhist countries such as Thailand (Lefferts, 2017; Malikhao, 2017), Myanmar (Foxeus, 2019, 2020), Sri Lanka (Holt, 2017; Abhayawansa, 2022), Cambodia (Hansen, 2017; Harris, 2017), and Laos (Lefferts, 2017; Sorensen and Nielsen, 2018; Ladwig and Rathie, 2020). It is important to note that Theravāda Buddhism continues to thrive in minority communities where the religious belief systems cannot be easily understood within the confines of doctrinal or institutional Buddhism in their individual countries. Buddhist tradition operates not only as a set of beliefs but also as part of a culture from which individuals derive their collective identity (Edwards, 2018). Recognizing that identities of collectives are constructed continuously, this understanding enables individuals to define their collective identity within a greater context (Cassaniti, 2025).

The Bulang ethnic community in Xishuangbanna, Yunnan, Southwest China, offers an important case for examining this relationship between Buddhism and collective identity. With their affiliation with Theravāda Buddhism, the Bulang have managed to maneuver themselves amid the challenges posed by the dynamics of modernity, state intervention, economic development, and generational differences to preserve their faith practices (Ocha, 2023; Wenen, 2024). The religious practice of the Bulang people occurs within a ritualized setting at temples, where monks officiate ceremonies, perform merit-making actions, tell stories, and participate in society at large. Temples, stupas, and shrines in this regard serve as venues for rituals and ethnic identifiers. This scenario brings into view the importance of Phra Somdet Aggamuni lineage.

Based on the literature review at present, one can deduce that Buddhism offers positive contributions in terms of social solidarity, ethics, and cultural perpetuity in a dynamic social environment (Darlington, 2019; Li and Wang, 2023; Krause, 2024). Other literature works have been written on transnational Buddhist network formations, adaptation to religions, and the responses of local Buddhist groups to modernity (Stapleton and Tao, 2022; Zhang et al., 2023). Despite these findings, there is inadequate knowledge about the Bulang case study, specifically in terms of understanding ethnographically the interactions of religion memory, monasticism, sacred landscape, and ritual performance in articulating collective identity. Previous works related to Theravāda Buddhism among ethnic minorities have tended to focus on issues of institutional growth, religious practice, and cultural protection without much emphasis on the exact dynamics involved in negotiating their identity.

This study addresses that gap by examining the Bulang community’s self-driven religious and cultural renewal through the framework of Theravāda Buddhist practice. It does not treat Humanistic Buddhism as an inherent or emic identity of the Bulang community. Rather, selected insights from Humanistic Buddhist thought are used as an analytical lens to interpret how Buddhist teachings are embedded in daily life, moral cultivation, communal solidarity, and cultural continuity. This distinction is important because the Bulang religious tradition is rooted in Theravāda Buddhism. At the same time, the language of Humanistic Buddhism is employed here only to clarify how Buddhist values are lived, localized, and socially enacted within the community. Accordingly, this article investigates how the Bulang community constructs and sustains collective identity through Theravāda Buddhism in the context of modernization, state regulation, and socio-economic change. It asks two main research questions:

1. How does the Bulang community mobilize Theravāda Buddhist practices, sacred biography, and religious infrastructure to construct and negotiate collective identity?
2. How do the Sangha, traditional elders, laypeople, and younger community members participate in sustaining the continuity of Theravāda Buddhism within the Bulang ethno-tradition?

Discussion of these issues provides insight into the sociological study of religion as well as Buddhist studies because the minority group has managed to use its religious memories, monastic tradition, sacred spaces, and practices to preserve its identity and culture in light of an ever-changing environment.

## Literature review

### Theravāda Buddhism and the construction of Bulang collective identity

Ethnic minorities’ collective identity is neither an inherited entity nor merely a static one; rather, it is a dynamic process whereby memory, ethics, rites, and culture are formed and asserted. The Buddhist tradition among the Bulang ethnic minority group in Xishuangbanna serves as the core construct for understanding, ordering, and transmitting their ethnic identity. Buddhism serves not only as a system of beliefs but also as the grammar of morality for the Bulang people to understand ideas of suffering, merit, discipline, social obligation, and continuity. Within such a framework, religion serves as a tool used in shaping one’s identity by tying up morality to one’s identity especially when faced with situations where modernity, government interventions, economic restructuring, and cultural influences play their roles (Cassaniti, 2025; Ocha, 2023; Wenen, 2024).

In the case of the Bulang community, it becomes evident that Theravada Buddhism is not just a set of beliefs, but an ethno-religious construct, which means that Buddhist ideology, practices, monks’ teachings, and local culture are combined with the identity of the people and their way of inscribing themselves as a minority population. It is important here since modernity is not only destructive towards traditions, but it requires communities to interpret their traditions as sources of resilience. In the case of the Bulang community, being Buddhist means not only preserving their cultural heritage but also employing strategies of ethnic survival and identity recognition through religion.

### Aggamuni, sacred biography, and soft religious infrastructure

The particularity about Bulang Theravāda Buddhism lies in the symbolic significance attached to the figure of Phra Somdet Aggamuni who is known by name of Aggamuni in local lore (Wenen, 2024). In that respect, the biography of this personality cannot be seen only as the life story of a revered Buddhist monk. It can be rather viewed as a sacred story through which the religious past of the community, its morals, and ethnic identity can be intertwined. The practice of preserving and ritually enacting the biography of Aggamuni represents, therefore, the act of cultural retrieval whereby the community uses not just any religious figure for remembering but turns into advantage his life for gaining symbolic significance. Through oral narration, ritualized speech, and storytelling, Aggamuni becomes the source of moral inspiration concerning self-restraint, renunciation, mercy, persistence, and dedication to the Dharma.

The hagiography may therefore be seen as an instance of what one might call “soft” religious infrastructure. As opposed to hard religious infrastructures like temples, stupas, and shrines that physically manifest the identity of Buddhism through concrete architectural forms, soft religious infrastructure acts through memory, storytelling, exemplarity, and affective attachments. In the case of the Bulang, Aggamuni constitutes the inner frame of reference for devotion. Through the lived experience of this figure, the community locates the Theravāda tradition in their own historical and cultural context and thus owns it as something that belongs to their own culture even though the doctrine itself is wider in scope than the village.

### Sangha, religious infrastructure, and the continuity of Bulang ethno-tradition

The Sangha has an important role to play in ensuring the survival of Bulang Buddhism. Monks act as spiritual advisors, ritual practitioners, role models for ethical behavior, and holders of Buddhist knowledge. Their authority operates via guiding practices, ritual practices, monastery training, and the utilization of Buddhism in their everyday practices. In this regard, the Sangha serves as the link between doctrine and laypeople, giving ethics a religious element while ensuring that the Bulang community has something to emulate from them. Moreover, the sustainability of Buddhism within the Bulang community relies on the contributions of both monks and lay practitioners, including women and youths, who help in the performance of rituals, telling stories, going to monasteries, and teaching Buddhist traditions (Puntarigvivat, 1998; Schedneck, 2021; Langgapin et al., 2024).

Indeed, the role of religion in sustaining this process is achieved through the formation of collective identity via ritual action in spatially constructed forms. In the building of temples, stupas, shrines, and other places for ritual practices, more than just religious acts are taking place; there is the manifestation of cultural validity and the reinforcement of community cohesion via the expression of collective identity. The creation of the economic, agricultural, and tourist resources gives the Bulang the means to form religious institutions that will convey their financial power and religious and ethnic identity. There have been studies done in relation to Bulang Buddhism (Stapleton and Tao, 2022; Zhang et al., 2023); however, these studies fail to explain the relationship between Buddhist doctrine, Sangha authority, hagiography, religious infrastructure, and lay involvement in the context of identity construction. To this effect, this study will address this gap by looking into the processes involved in the practice of Theravāda Buddhism within Bulang society.

## Methodology

### Research design

The ethnographic methodology was employed in this study to explore how the Bulang society establishes and sustains their collective identity via Theravāda Buddhism within Laoman’e village, Yunnan, China. This study was purely based on qualitative research methods without making use of questionnaires and other forms of statistics. Instead, it relied on in-depth fieldwork, participant observation, and semi-structured interviews with 10 purposively selected participants, including monks, traditional elders, and community members who were directly involved in religious practice, ritual activities, cultural preservation, and community life. It is selected because of the intention to understand religious experience in life, the monastic and lay interrelations, biographies of the sacred people, ritual participation, and religious institutions as social means of identity construction. Investigating how Buddhist doctrines, authority of the Sangha, memory of Aggamuni, temples, stupas, and ritual practice are used in the daily context of the community’s members, we present an ethnographic description of how this particular group manages to preserve the ethno-religious tradition amid modernization processes and governmental policies.

### Participants

In conducting an investigation into Theravada Buddhism in an ethnic minority group, a multidimensional methodology that takes into account field observations, interviews, and local religious history is important. The practice of Buddhism is learned not just from the textual knowledge but through ritual activities, the interaction between monks and lay followers, the creation of sacred space, and storytelling. In line with the ethnographic paradigm, the current study utilized a purposive sampling design as opposed to randomization. The research goal was not numerical generalizability but gaining in-depth understanding from people actively involved in sustaining Bulang Theravada Buddhism. A total of 10 participants were involved in the study, consisting of monks, traditional elders, lay community members, and younger participants in Laoman’e village, Xishuangbanna, Yunnan, China. The individuals selected for this study have been involved in the performance of religious rituals, temple activities, and cultural preservation. Additionally, they have also participated in the passing down of the life story of Aggamuni orally. The interviews conducted with monks focused on the doctrinal advice, authority of rituals, and the role of the Sangha, those interviewed with elders provided information about memories of the past generations, and interviews and informal discussions with laypeople and young people provided knowledge regarding the maintenance of the Buddhist identity. The participant composition is presented in Table 1.

**Table 1:** Interview participants and fieldwork contexts.

Participant group	Fieldwork context	Number of participants	Main relevance to the study
Monks/Sangha members	Laoman’e Buddhist temple, ritual activities, monastic guidance	3	Provided perspectives on Theravāda teachings, ritual leadership, Sangha authority, and the transmission of Buddhist values.
Traditional elders	Village-based religious and cultural settings	2	Provided oral histories, local memory, and accounts of Aggamuni’s significance in Bulang Buddhist identity.
Lay community members	Temple ceremonies, stupa-related activities, communal rituals	3	Provided perspectives on lay participation, merit-making, temple support, and the role of religious infrastructure in community cohesion.
Younger community participants	Village religious activities and intergenerational cultural transmission	2	Provided insight into youth engagement with Buddhist tradition, cultural continuity, and adaptation to modernization.
Total		10	

### Data collection

Data were collected through in-depth ethnographic fieldwork, participant observation, and semi-structured ethnographic interviews in Laoman’e village, Xishuangbanna, Yunnan, China. In line with the tradition of using ethnography for studying Theravāda Buddhist communities (Tambiah, 1970; Van Esterik, 1985), religion in the study is not viewed simply as doctrine, but rather as a way of life that is rooted in ritual performance, sacred geography, interplay between monastics and lay practitioners, oral tradition, and communal practices. Laoman’e was selected as the main field site as the place where Theravāda Buddhism was still strongly associated with ethnicity, ritual practices of the village, temple-based activities, and local religious stories. Observations on the field paid special attention to Buddhist rituals, temple/stupa practices, interaction between monks and lay people, and communal activities related to religion.

In total, 10 informants were selected using the method of purposive sampling, which includes monks, elders of the traditional society, people from the community as well as younger generations involved in the practice of Bulang Buddhism. Interviews were used to gather information about the Theravāda Buddhism doctrine, participation in rituals, the role of Sangha, the importance of religious infrastructure, as well as the person of Aggamuni in the community’s religious life. Observational methods were used to verify data obtained from the interviews by analyzing the actual use of religious meaning in practice, rather than merely through language. Local myths and oral narratives were also studied with regard to the temple, stupa, and religious biographies as tools for building ethnic-religious identity. With the help of interviews, observation, and field notes, qualitative data can be collected regarding how the Bulang community uses Theravāda Buddhism to maintain its cultural identity and strengthen its ethno-religious identity.

### Data analysis

Thematic analysis was applied to semi-structured interview transcripts, field notes, and participant observation records collected from 10 purposively selected participants. This particular methodology was adopted owing to the fact that the research was focused on exploring patterns of meaning in the lived religion of the Bulang people. Thus, no attempts were made to quantitatively measure the variables under analysis in any form. The interview data collected from various participants was transcribed, validated, and analyzed using the techniques recommended by Braun and Clarke (2006), Bryman (2008), and Creswell and Creswell (2023). Specifically, the analysis centered on the participants’ discourse pertaining to Theravāda Buddhism as well as the expression of the discussed meanings in rituals and everyday life.

In light of the research questions and the study’s aims, the fieldwork data were categorized into three main analytical dimensions. The first dimension concerned the construction and negotiation of Bulang collective identity through Theravāda Buddhist teachings, moral discipline, ritual participation, and responses to modernization and state regulation. The second dimension looks at the functions of various figures in the community that contribute to sustaining religious continuity through Sangha power, oral tradition, and the life history of Aggamuni. In the third dimension, the functions of religious facilities such as temples, stupas, shrines, and collective rituals are examined as tools for culture preservation and ethnoreligious identity. The analysis of each dimension is made from the perspective of the main issue addressed in the research, namely, the role of Theravada Buddhism in constructing collective identity for the Bulang community.

## Result

### Religious infrastructure and the public construction of Bulang Buddhist identity

Before proceeding to the fieldwork findings, it is necessary to clarify the position of Theravāda Buddhism in Laoman’e village. For the Bulang people, Buddhism is not just a set of religious principles but rather an ideology that helps to define them as a unique ethno-religious community. Temples, stupas, shrines, monasteries, ritual places, and ceremonies can all be used to signify this unique identification. They do not simply accommodate religious activities; they materialize the community’s claim to continuity, legitimacy, and belonging. In response to pressures associated with modernization, tourism, government regulation, and socioeconomic change, rebuilding and maintenance of Buddhist architecture have become important ways for the Bulang people to ensure their belief in the continued social relevance and cultural legitimacy of Buddhism among them. Evidence from the field suggests that there has been a direct correlation between building of Buddhism-related architecture in Laoman’e and the community’s attempt at translating material prosperity into religious legitimacy. Some respondents even described the creation and restoration of temple and stupa construction not as normal architectural activity but as an expression of faith and cultural identity:

*Elder (A):* Building and repairing the temple is not only for worship. It shows that our village still respects Buddhism and has not forgotten its ancestral traditions.

*Lay participant (A):* When we contribute to the temple, we feel that we are doing something for the Buddha, for the monks, and also for the Bulang people. The temple belongs to all of us.

*Elder (B):* Investing in our temples is a way to show our commitment to Buddhism and to gain respect from neighboring communities.

*Lay participant (B):* When I see the stupa, I am reminded of the Buddha’s teachings, and it strengthens my commitment to practice.

*Youth participant (A):* The temple is where we see our tradition directly. We may not understand everything from books, but when there is a ceremony, and people gather, we know this is part of being Bulang.

**Figure 1:**
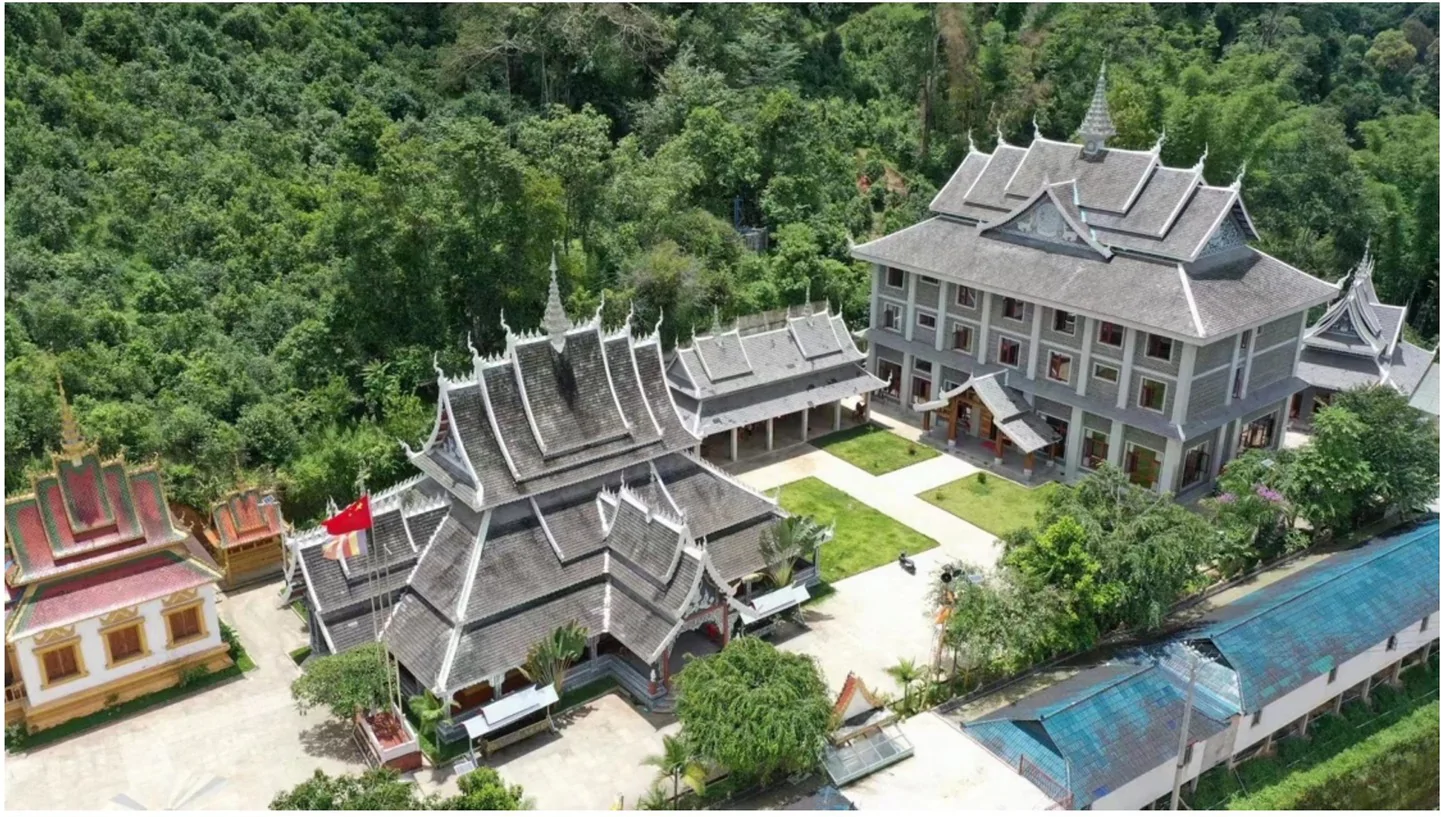
Overview of the major Buddhist wat/temple complex in Laoman’e Bulang Village, sponsored and built by Bulang villagers in 2023–2024.

There are several points worth considering based on the two passages. Firstly, the building and renovation of Buddhism-related structures in Laoman’e can no longer be simply explained in terms of revival and modernization of religion or architecture. Instead, what emerges from the participants’ discourse is a picture of how the Bulang utilize the means at hand and turn them into symbols of legitimacy in a society in which the influence of modernization, tourism, and even state policy has changed the sociocultural environment of the minority ethnic villages. The temple complex becomes, thus, an indication of the Bulang people’s efforts to prove that they are not simply adapting to changes but affirming themselves as Buddhists in the midst of these changes (see Figure 1).

Furthermore, the religious architecture of Laoman’e is active on three interconnected planes, including ritual, social, and political-symbolic. In terms of the ritual dimension, the temples and stupas create the material space for worshiping, offerings, and rituals as well as interaction among the monastic and lay community members. Socially, such temples serve as a framework for collective work, giving, sharing memories, and multigenerational involvement. Politically, they enable the visualization of Bulang Buddhism to the outside world. As a result, the temple complex is not simply a location for the manifestation of identity in a posterior manner but also an important stage of its formation and demonstration. The community does not simply “have” a Buddhist identity and then build temples. Rather, the act of building, maintaining, funding, visiting, and ritualizing these spaces is itself part of the process by which Bulang Buddhist identity becomes durable.

Third, the field data provide evidence against the common belief that modernization inevitably undermines the practices of minority religions. Modernization leads to both negative and positive consequences for the Bulang in this situation. First, it entails the emergence of new factors, such as tourism, economic stratification, the visibility of the state, and the transformation of the aspirations of young people. Second, it provides opportunities that allow this community to reconstruct its temples, organize rituals, and increase the role of Buddhism in its public life. Hence, religion continues in Laoman’e not just by preserving traditional culture but also by revitalizing it under different circumstances.

Moreover, and equally important, the religious infrastructure helps to explain the process through which the Bulang people build their identity together. It is true that the temple is symbolically owned by monks and that the importance of this place is also not restricted to older followers only. According to the stories narrated by participants, different groups of individuals such as monks, elders, laypeople, and younger people interpret the same place with varied but intersecting significance. In particular, while monks see it as a place of education and ritual order, elders find it to be a place where ancestors are remembered, laypeople see it as a place of merit-making and collective duty, and youth see it as a tangible sign through which Bulang culture can be identified.

### Aggamuni, sacred biography, and soft religious infrastructure

The second major finding involves the importance of Phra Somdet Aggamuni, who is sometimes called Meghamuni in certain oral traditions in the area. To prevent terminological confusion, this research paper uses the name Aggamuni to describe the individual while noting that Meghamuni refers to the local name for him in a sacred narrative. It would be incorrect to consider the significance of Aggamuni for Bulang Buddhism through the lens of remembrance of one revered monk; rather, his story is viewed as a form of sacred speech that links religion, community memory, ethical conduct, and ethnicity together. The story of Aggamuni, according to the oral traditions shared in the fieldwork, is seen not as something from the past but as a living presence whose teachings serve as a guide for maintaining the Buddhist tradition.

Aggamuni’s story can be considered a symbol of connection between the Bulang community and its history. For example, participants described his figure as a link to Buddhist ethics such as discipline, renunciation, compassion, perseverance, and dedication to the Dharma. Additionally, the oral tradition describes his life as a legacy worth telling the younger generation about because it allows people to trace back the origins of their religion:

*Elder (A):* We remember Aggamuni because he represents the Buddhist path of our people. His life teaches us discipline, patience, and respect for the Dharma.

*Monk (A):* For the Bulang community, Aggamuni is not only a monk from the past. His example is still used to teach people how to live according to Buddhist values.

*Elder (B):* The story of Meghamuni must be passed down to children. If they know his story, they will understand where our faith and our tradition come from.

*Lay participant (A):* When we come to the shrine, we do not only remember a person. We remember the teaching, the virtue, and the connection between Buddhism and our village.

*Youth participant (A):* I heard about Meghamuni from elders and monks. His story makes me understand that Buddhism is not only something we see in ceremonies, but something connected to our history as Bulang people.

**Figure 2:**
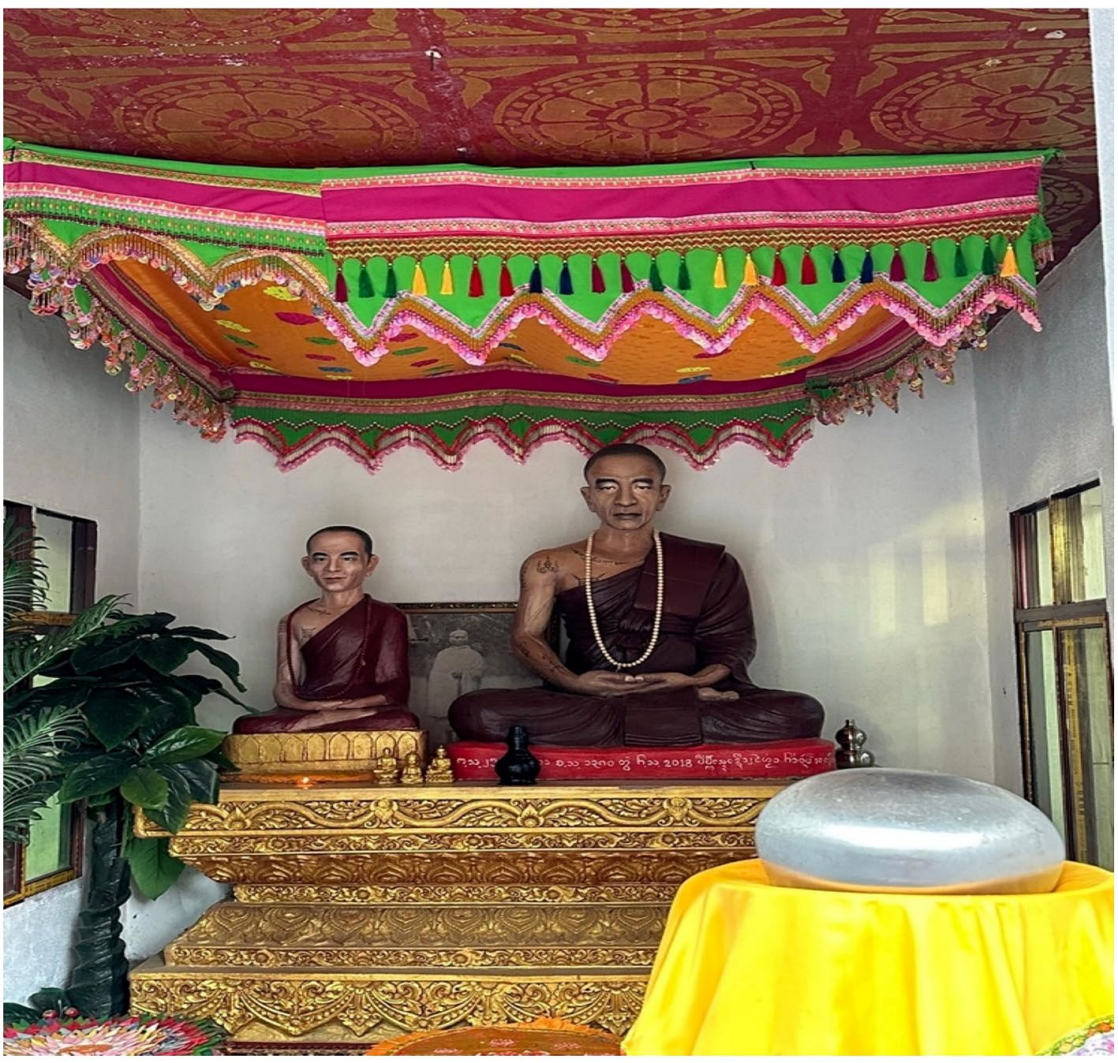
The recently installed shrine of Phra Somdet Aggamuni, a legendary Bulang Buddhist monk widely venerated in Xishuangbanna, according to oral accounts preserved in Laoman’e village.

These narratives raise several important points. First, Aggamuni’s biography operates as a moral archive for the Bulang community. Throughout his lifetime memory, Buddhist virtues are not just taught through concepts, but through real-life models of those virtues. Such virtues like discipline, renunciation, compassion, endurance, and commitment to the Dharma become understandable because they belong to someone in the religious memory of the Bulang people. In this sense, holy biography not only preserves knowledge of the past but shapes the ethical imagination of the present. Through the model of Aggamuni, the community comes to know what kind of Buddhist behavior should be respected, what kind of life should be copied, and why Buddhism continues to matter to them (see Figure 2).

Secondly, the fact that both Aggamuni and Meghamuni are referred to by the interviewees cannot be viewed simply as a matter of inconsistent vocabulary usage. It actually reflects the way memory can be passed on within an oral culture. The importance of naming does not lie in its uniformity; rather, what is important is whether the person has earned the community’s recognition of his or her moral status. The difference between Aggamuni and Meghamuni highlights the fact that memory is transferred through storytelling and ritual recitations by elders and villagers.

A final point is connected with the shrine of Aggamuni. First of all, it shows that religious infrastructure in the Bulang setting implies both material components and those of the narrative kind. In particular, temples, stupas, and shrines represent tangible sacred places whereas Aggamuni’s life represents the so-called soft religious infrastructure. The concept under discussion includes stories, memories, rituals, testimonies, emotions, and the like that support the community’s inner moral infrastructure by telling about the significance of sacred buildings, the importance of continued rituals, and reasons why future generations should embrace Buddhism. Without a soft religious infrastructure, temples would only mean brick constructions; thanks to Aggamuni’s sacred biography, shrines have come to symbolize memory, legitimacy, and morality.

However, the biography is also crucial since it allows the Bulang community to localize the Theravāda Buddhist religion. Even though Buddhism is traditionally considered trans-local, for the Bulangs of Laoman’e it acquires meaning due to their own memory and local sacred characters. Thus, thanks to Aggamuni, Buddhism no longer appears to be a foreign religion; rather, it becomes part of the Bulangs’ history, moral heritage, and collective property. Hence, the shrine is not limited to memorializing the monk; through Aggamuni, it turns biography into the community’s public memory and then into its very identity. Such a conclusion is especially valuable since it means that besides traditional doctrines and institutions the continuation of the Buddhist tradition among the Bulangs cannot do without the remembrance of individual people. Aggamuni’s sacred biography serves as the inner scaffold of identity; it creates ties with the past, makes Buddhist practices emotionally charged, and offers an example of morality.

### Sangha, lay participation, and ritual continuity

The third major finding concerns the relational structure through which Bulang Theravāda Buddhism is maintained. Before presenting the fieldwork data, it is important to note that religious continuity in Laoman’e village does not depend on a single actor or institution. The Sangha has a vital role in its position as the keeper of Buddhist teachings, ritual authority, and morality. Nevertheless, the sustainability of Bulang Buddhism comes from the involvement of the elders, lay followers, women, young people, and ritual practitioners. With this observation, the propagation of Theravāda Buddhism among the Bulang people is not simply about teaching from within the monastery, as there is still an interlinking of teaching, ritual, memory, donation, and labor through the social network.

From field data, it becomes evident that the Bulang believe the monks to be their teachers, guardians, and moral examples. It was consistently highlighted by the interviewees that the Sangha acts as a spiritual guide, not only in ritual practice but even in daily communal activities. At the same time, they stressed that it would not have been possible without the involvement of the laypeople and elders:

*Monk (A):* The Sangha has the responsibility to teach the Dharma and guide the people. If the community does not understand the teaching, the temple will be no more than a building, not a place of practice.

*Elder (A):* The monks are our teachers and protectors. They guide us to live a good life and follow the path of the Buddha.

*Lay participant (A):* We support the monks because the temple cannot continue without the people. We prepare offerings, help with ceremonies, and contribute what we can to the temple.

*Elder (B):* We pass down the story of Meghamuni to our children so they can understand the roots of our faith and our tradition.

*Youth participant (A):* During ceremonies, we learn from watching the elders and monks. We may not know all the teachings yet, but by joining the rituals, we understand that Buddhism is part of our Bulang identity.

**Figure 3:**
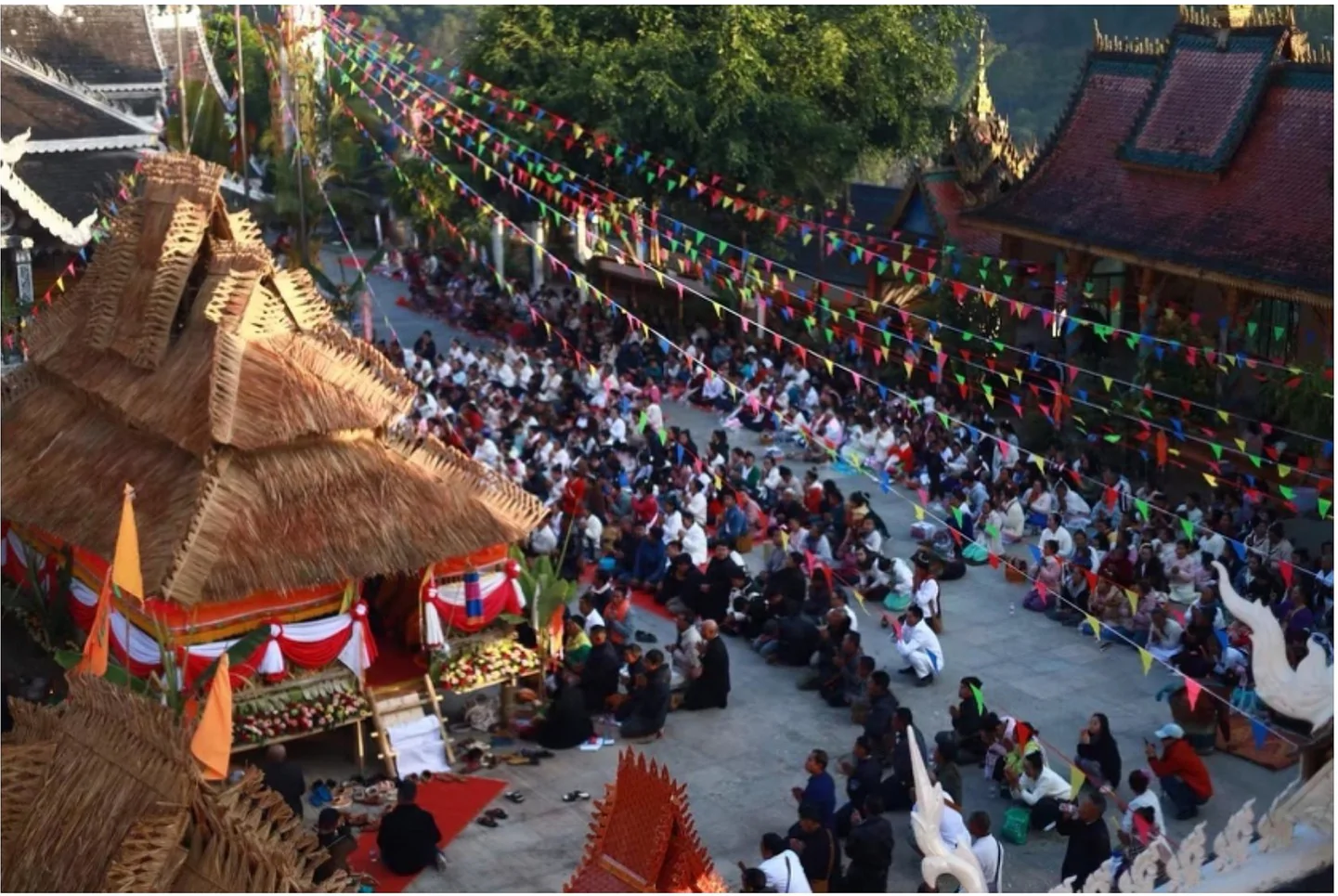
The gathering of Laoman’e Bulang people in October 2025 to celebrate the completion of Buddhist Vassa, also known as the Kaṭhina ceremony.

These quotations and field observations highlight a number of aspects related to this issue. First, although the Sangha represents a source of continuity concerning teachings and rules of conduct, the social influence exerted by the monastery can be achieved only if the lay community is involved in the process. Monastics deliver lessons, provide guidance, and organize various rituals; nonetheless, their continuation is impossible without the active participation of community members. Hence, the monastic community cannot be regarded as the only driving force within the temple as the lay community contributes to the functioning of the religion by making financial contributions, preparing offerings, organizing ceremonies, maintaining sacred spaces, and introducing new generations to the rituals. Without lay people, monastic authority would remain an integral part of religion without having any social significance (see Figure 3).

Second, it would be incorrect to refer to lay community members as passive receivers of monastic knowledge. On the contrary, they can be seen as agents contributing to religious practice by taking part in ceremonies. By means of performing rituals, preparing offerings, cleaning the temple, and organizing celebrations, lay community members make Buddhism a part of their life. Therefore, it is fair to state that the continuity of Bulang Theravada Buddhism relies not only on the contribution of the Sangha but also on the interaction between both groups. The elders act as intermediaries in the link between religious learning and family-based education due to their ability to preserve as well as explain history to the younger generation. Through teaching history about Aggamuni, explaining the rites and guiding the junior members of the community, the elders establish a connection between history and practice, thus making it possible for the young Bulangs, who might not know much about the religion previously, to learn about Buddhism.

In addition, the practice of the Kaṭhina ceremony represents an example of how rituals transform individual religion into a collective experience. Rituals transcend the regular annual Buddhist celebration marking the end of the Vassa season; they turn into the public performance of the mutual commitment of monks and followers. By way of offerings, participation in the process, ritual activities, and involvement, the community reasserts its relationship with the Sangha and reaffirms itself as Theravādin Buddhist. The significant involvement shows that Buddhism is very present in the community and continues organizing the rhythms of life and social relationships of the villagers as well as the idea of ethnicity. Therefore, ritual performance is not only the expression of identity but also its embodiment.

Furthermore, the participation of youth in rituals implies both continuity and instability of the local Buddhist tradition. While the participation of young members shows that Buddhist identity is transmitted from one generation to another, at the same time, the necessity of their relying on observation rather than doctrine suggests that the continuation of Buddhist tradition cannot be taken for granted. The future development of Buddhism among the Bulang depends on the ability of rituals to contribute not only to the preservation but also to the deeper knowledge of doctrines, traditions, and the perpetuation of sacred memory.

As can be seen from the presented findings, continuity in Bulang Theravāda Buddhism is relational. Continuity takes shape when there is an interaction between religious authority, lay devotion, elder knowledge, youth inclusion, sacred biography, and religious infrastructure. Religious authority is provided by the Sangha; memory and knowledge are supplied by the elders; laypeople offer material assistance and ritual work; finally, youth adds an element of future continuity to the process. In this regard, it is safe to claim that the Kaṭhina ritual is much more than just a ritual; it is a concentrated expression of the Buddhist identity of the Bulang community through teaching, offering, memory, participation, and belonging. To conclude, the presented fieldwork data clearly show how the Bulang community develops its collective identity using three interconnected means. The first one is religious infrastructure which expresses the religious dedication and public identity of the community. The second means is the sacred biography of Aggamuni which works as the soft religious infrastructure and serves as a basis for moral memory and intergenerational transmission of the knowledge. Finally, the third means includes both the Sangha and laypeople, who together perpetuate the Theravāda Buddhism by means of rituals, temple support, and communal participation.

## Discussion

Accordingly, the results of this investigation suggest that Theravāda Buddhism serves as a central device by which the Bulang people construct and negotiate their collective identity in the Laoman’e village. Contrary to the idea of Buddhism being understood as merely a system of religious doctrine or an individual matter of faith, the Bulang utilize Buddhism as an ensemble of practices, institutions, rituals, and memory of history that serve to sustain their culture. Such results correspond to previous scholarly works that focus on the significance of Buddhism in terms of ethical behavior, social solidarity, and collective identity within Theravāda communities (Puntarigvivat, 1998; Schedneck, 2021; Langgapin et al., 2024). Nevertheless, the uniqueness of the present study lies in the fact that Buddhism not only serves as the foundation for ethical living but becomes a tool that allows a minority ethnic community to affirm its cultural existence in the context of modernization, government control, tourism industry development, and socio-economic shifts.

Firstly, this study shows that religious infrastructure plays a crucial role as an important medium of identity construction. Temples, stupas, shrines, monasteries, and ritual spaces cannot be considered merely as physical locations of Buddhist worship. Rather, they represent physical markers of Buddhist community and serve as tangible forms of memory for the community members. According to field work findings, Buddhist infrastructures become means by which Bulang can transform economic resources into symbolic legitimation. In this sense, the study confirms earlier arguments about the central role of religious sites and heritages in the process of social identification (Di Giovine & Choe, 2020; Thouki, 2022; Burchardt and Ural, 2024). However, unlike other approaches that consider religious sites primarily as heritage objects, the current study illustrates that sacred places of the Bulang community continue performing important social functions.

Indeed, this discovery adds another dimension to the assumption that the process of modernization necessarily leads to the weakening of minority religious traditions. In Laoman’e, modernization results in mixed impacts. First of all, it puts pressure on the local population by means of tourism, state presence, changes in markets, and changing aspirations of the younger generations. At the same time, modernization creates new economic opportunities to rebuild temples, conduct ceremonies, and create a more prominent Buddhist presence. Therefore, in the example of the Bulang people, one can see that religion is not an old and fading phenomenon, but a deliberately renewed tradition adjusted to new circumstances.

The second important contribution of this research relates to the analysis of Phra Somdet Aggamuni, whose life history is also referred to in some local oral tradition as Meghamuni, and his importance in the context of the Bulang society. In contrast to previous research on the topic of Buddhism in local contexts that mostly focuses on doctrines, institutionality, rituals, and religion networks (Friedland, 2021; Qadir and Alasuutari, 2022; Zhang et al., 2023), the current study shifts attention from those aspects, expanding the analysis and taking sacred biographies into consideration. Thus, the current study regards the phenomenon of sacred biography as an integral part of religious infrastructure. Within the context of the Bulang, the sacred biography of Aggamuni is not just a record of events, but rather a source of moral values such as discipline, renunciation, mercy, perseverance, and compliance with Dharma.

The idea of soft religious infrastructure can be useful in analyzing the case of Bulang. While temples and stupas form visible infrastructure, sacred biography represents narrative and affective infrastructure which includes stories, virtues, rituals, the testimony of elders, and reverence among others. In this context, therefore, the difference between Aggamuni and Meghamuni cannot simply be seen as an inconsistency. Instead, it can be taken to represent the existence of a live story that has been handed down from generation to generation. The transmission of sacred power in the context of a live story is achieved through both written and oral discourse. Buddhism is embedded in the history of Bulang people through the story of Aggamuni. It is no longer alien to their consciousness.

Thirdly, this study offers a relational understanding of the dynamics of religious continuity. The findings affirm the critical importance of the Sangha as a source of authority regarding doctrine, ritual leadership, and morality. Such findings align with previous studies that emphasize monks’ importance as moral models and intermediaries between Buddhism and lay society (Puntarigvivat, 1998; Schedneck, 2021; Langgapin et al., 2024). At the same time, this work demonstrates that the religious continuity of Theravāda Buddhism cannot be reduced to the mere presence of monastic authority. Religion is sustained through a relational process involving monks, elders, lay people, women, youth, and ritual performers. Whereas monks provide religious guidance, lay people offer logistical support, help organize the rituals, give donations, prepare food, maintain the temple, and engage in ritual activities themselves. The responsibility to preserve the community’s memory is fulfilled by elders, while youth are representatives of the community’s precarious but crucial future.

This relation dynamic is clearly observed in the Kaṭhina ceremony. The ritual not only represents the annual Buddhist ritual held at the end of Vassa, but a performance through which the community expresses its affiliation with Theravāda Buddhism. As such, the ritual serves as a reminder of the importance of the Sangha and the community’s identity, as expressed in the ritual itself. Importantly, ritual, according to this understanding, does not just express an identity that already exists, but helps build and sustain it by bringing different individuals together in the performance of the shared experience of religion.

The above results simultaneously suggest the existence of a crucial tension that will define the future of Bulang Buddhist identity. While the involvement of younger Buddhists shows the continuation of the tradition, it is important to note that their use of rituals without full understanding of doctrines does not mean that there will necessarily be continuity in the future. The fate of Bulang Theravāda Buddhism depends on the development of ritual involvement into religion, i.e., gaining deeper knowledge and the relevance of sacred memory among young people in the face of educational, migration, tourist, technological challenges, and changing career ambitions. This tension forms the basis of negotiating modernity among Bulangs.

In general, the current research has enriched the sociology of religion, Buddhist studies, and minority identity studies by showing that the identity of a community can be formed through the interplay of material, narrative, and relational mechanisms of religion. First, the temple and stupa make Bulang Buddhism materially present. Secondly, Aggamuni’s holy life provides sacred memory and ties Buddhism to the history of the Bulang people. Third, the interaction between the Sangha, elderly monks, laymen, and young people ensures continuity of religion through rituals, donations, teaching, storytelling, and ritual attendance. This research has not been limited to considering religion only as culture. For Bulangs, Theravada Buddhism is a functional identity formation system that allows preserving traditions, negotiating modernity, providing legitimacy, and maintaining collective identity.

## Conclusion

Accordingly, this study proposes that the Bulang collective identity is shaped and sustained through their practices of Theravāda Buddhism through the combination of Buddhist doctrine, religious infrastructure, sacred biography, and collective participation. Specifically, it is found that in Laoman’e village, Theravāda Buddhism is more than a belief system or personal spirituality but a moral, cultural, and social system that allows the Bulang to construct their sense of community and culture, preserve their memory and traditions, and engage with modernity, state control, tourism, and socio-economic transformation. The temples, stupas, shrines, and ritual places are symbols of their devotion to the Buddhist way of life and the recognition of their Bulang identity. Moreover, it can be seen that there is also the notion of soft religious infrastructure in this community in the form of Phra Somdet Aggamuni (or Meghamuni), a saintly figure who has become the focus of the community’s moral memory and authority passed down from generation to generation. From these findings, it is evident that the continuity of Theravāda Buddhism in this community is based on the interaction among different members of the community as well as their collective activities. More specifically, in the case of this small ethnic community, the Sangha serves as doctrinal authority; the elders maintain oral history; the laity maintains Buddhism in everyday life; and the young members of the community are the future of this tradition through their participation in rituals such as Kaṭhina. These findings imply that the preservation of Buddhism within the community is not only about the continuation of an inherited tradition but also about its embodiment, narration, and re-construction as a strategy of cultural survival.

## Data Availability

The data used in the current study contain private details concerning the human subjects involved and therefore cannot be shared publicly because consent was not granted for public use. Anonymized sections relevant to the research findings have been included in the article. Further inquiries can be directed to the corresponding author.
